# An Actigraphy-Based Validation Study of the Sleep Disorder Inventory in the Nursing Home

**DOI:** 10.3389/fpsyt.2020.00173

**Published:** 2020-03-13

**Authors:** Gunnhild J. Hjetland, Inger Hilde Nordhus, Ståle Pallesen, Jeffrey Cummings, Rochelle E. Tractenberg, Eirunn Thun, Eirin Kolberg, Elisabeth Flo

**Affiliations:** ^1^Department of Clinical Psychology, Faculty of Psychology, University of Bergen, Bergen, Norway; ^2^City Department of Health and Care, Bergen, Norway; ^3^Norwegian Institute of Public Health, Bergen, Norway; ^4^Department of Behavioral Sciences in Medicine, Faculty of Medicine, University of Oslo, Oslo, Norway; ^5^Department of Psychosocial Science, Faculty of Psychology, University of Bergen, Bergen, Norway; ^6^Norwegian Competence Center for Sleep Disorders, Haukeland University Hospital, Bergen, Norway; ^7^Department of Brain Health, School of Integrated Health Sciences, University of Nevada, Las Vegas, NV, United States; ^8^Cleveland Clinic Lou Ruvo Center for Brain Health, Las Vegas, NV, United States; ^9^Collaborative for Research on Outcomes and –Metrics, Silver Spring, MD, United States; ^10^Departments of Neurology, Biostatistics, Bioinformatics & Biomathematics, and Rehabilitation Medicine, Georgetown University, Washington, DC, United States

**Keywords:** dementia, sleep, proxy-rating, actigraphy, nursing home

## Abstract

**Background:** Disrupted sleep is common among nursing home patients with dementia and is associated with increased agitation, depression, and cognitive impairment. Detecting and treating sleep problems in this population are therefore of great importance, albeit challenging. Systematic observation and objective recordings of sleep are time-consuming and resource intensive and self-report is often unreliable. Commonly used proxy-rated scales contain few sleep items, which affects the reliability of the raters' reports. The present study aimed to adapt the proxy-rated Sleep Disorder Inventory (SDI) to a nursing home context and validate it against actigraphy.

**Methods:** Cross-sectional study of 69 nursing home patients, 68% women, mean age 83.5 (SD 7.1). Sleep was assessed with the SDI, completed by nursing home staff, and with actigraphy (*Actiwatch II, Philips Respironics)*. The SDI evaluates the frequency, severity, and distress of seven sleep-related behaviors. Internal consistency of the SDI was evaluated by Cronbach's alpha. Spearman correlations were used to evaluate the convergent validity between actigraphy and the SDI. Test performance was assessed by calculating the sensitivity, specificity, and predictive values, and by ROC curve analyses. The Youden's Index was used to determine the most appropriate cut-off against objectively measured sleep disturbance defined as <6 h nocturnal total sleep time (TST) during 8 h nocturnal bed rest (corresponding to SE <75%).

**Results:** The SDI had high internal consistency and convergent validity. Three SDI summary scores correlated moderately and significantly with actigraphically measured TST and wake-after-sleep-onset. A cut-off score of five or more on the SDI summed product score (sum of the products of the frequency and severity of each item) yielded the best sensitivity, specificity, predictive values, and Youden's Index.

**Conclusion:** We suggest a clinical cut-off for the presence of disturbed sleep in institutionalized dementia patients to be a SDI summed product score of five or more. The results suggest that the SDI can be clinically useful for the identification of disrupted sleep when administered by daytime staff in a nursing home context.

**Clinical Trial Registration:** www.ClinicalTrials.gov, identifier: NCT03357328.

## Introduction

Sleep problems and disturbed nocturnal behavior constitute important aspects of the behavioral and psychological symptoms of dementia (BPSD) (1). In nursing homes, night-time wandering, confusion, and related behaviors can increase the risk of patient injuries, e.g., falling (2), and may cause disturbances for other residents. Such behaviors are also distressing and resource demanding for the staff (3).

As part of normal aging, characteristic changes in sleep and circadian rhythmicity take place. These entail a reduction in sleep duration and the proportion of slow wave sleep, as well as sleep fragmentation and an increase in the frequency and duration of daytime naps (4). Commonly, the sleep phase is advanced, implying that older people tend to experience sleepiness earlier in the evening and wake up earlier in the morning than desired. Also, the prevalence of some primary sleep disorders, such as sleep-disordered breathing, increases with age (5).

Sleep and circadian alterations are more frequent in patients suffering from dementia than in normal aging, and studies have provided estimates of disturbed sleep from 24% (6, 7) to 70% in dementia populations (8, 9). Brain systems involved in sleep and wakefulness are often increasingly affected as neurodegeneration progresses (10). Moreover, the causes of disturbed sleep in dementia are multiple, and factors such as inactivity, medications, and reduced exposure to social interaction and reduced daylight exposure are all associated with disturbed sleep (11–13).

Disruption of sleep and circadian rhythmicity have been associated with increased agitation (14), depressive symptoms (15–17), and cognitive impairment (15, 18) in people with dementia. In addition, disturbed sleep has been identified as an important cause of caregiver distress (19, 20) and of institutionalization of patients suffering from dementia (21–23). Detecting and treating disturbed sleep is of crucial importance in relation to improving behavioral and mood related symptoms, enhancing well-being, and reducing caregiver distress.

Assessing disturbed sleep in people with dementia is challenging, as self-report may be unreliable and, in many cases, unfeasible. Most et al. (24) demonstrated that even in the early and middle stages of Alzheimer's disease (AD), patients had more objectively measured sleep problems compared to healthy age-matched controls, however, the former group self-reported fewer sleep problems. Hence, clinicians and researchers often rely on proxy-rater instruments, where nurses or relatives answer on behalf of the patient. Unfortunately, research suggests that nursing home staff often provide unreliable and inaccurate reports of their patients' sleep when not using adequate instruments (25–27). For example, using the sleep items of the Cornell Scale of Depression in Dementia (CSDD) and the Neuropsychiatric Inventory (NPI) in a nursing home population, Blytt et al. (25) found that staff reported significantly fewer sleep problems than measured by actigraphy. Their study suggested that disturbed sleep may go largely undetected in the nursing home population when measured by staff rated instruments with only one or a few items. Meanwhile, using the comprehensive 21-items Circadian Sleep Inventory for Normal and Pathological States (CSINAPS), completed by nursing home nurses, Hoekert et al. (27) found only small-to-medium associations between actigraphy parameters and scale items and subscales. However, systematic observation of sleep behavior often requires that staff frequently or continuously observe each patient across several days (28, 29). Such time consuming and resource intensive assessments are not necessarily feasible in a nursing home context. Further, objective measures of sleep such as actigraphy are rarely used in clinical contexts due to the cost of the equipment and the time and skill needed to interpret the output. Thus, identifying a relatively short questionnaire that more accurately detect sleep problems in dementia populations has the potential of providing caregiving staff with a clinically important and more feasible tool.

To the authors' knowledge, the Sleep Disorder Inventory (SDI) (30) is the only short-form scale that exclusively focuses on evaluating sleep in dementia populations. Tractenberg and colleagues (30) have demonstrated appropriate convergent validity (i.e., significant correlations with actigraphy) in a group of home-dwelling participants suffering from AD. To date, the SDI has not been validated for use in the nursing home context, despite the need for clinically relevant and easy-to administer sleep assessment tools in these settings.

Accordingly, the aim of the current study was to evaluate the SDI in the nursing home context after adapting item wording accordingly. Specifically, we aimed to: (i) assess the adapted SDI's internal consistency, (ii) investigate the convergent validity of the adapted SDI against actigraphy, (iii) and suggest a clinical cut-off for disturbed sleep in nursing home patients with dementia.

## Materials and Methods

### Sample and Setting

This study used baseline data from a 6-months cluster-randomized placebo-controlled trial, evaluating the effectiveness of bright light treatment in people with dementia (the DEM.LIGHT trial, ClinicalTrials.gov Identifier: NCT03357328). The trial was conducted in Norway from September 2017 to April 2018. We invited the Department of Health and Care, City of Bergen, Norway, to participate in the study with eight eligible nursing home dementia units (e.g., nursing homes that were not involved in other trials or quality of care projects and that had an architecture that allowed for ceiling light installment). See Table 1 for inclusion and exclusion criteria.

**Table 1:** List of sample inclusion and exclusion criteria.

**Participants were eligible if they:**	**Patients were not included in the study if they:**
- were ≥60 years and in long-term care (>4 weeks) - had dementia in accordance with DSM-5 - had either sleep/circadian rhythm disturbances, BPSD as identified by NPI-NH, or severely reduced ADL function - provided written informed consent if the participant had capacity or, if not, a written proxy informed consent from a legally authorized representative	- were blind or may otherwise not benefit from light - took part in another trial - had a condition contra-indicated to the intervention - had an advanced, severe medical disease/disorder and/or expected survival less of than 6 months or other aspects that could interfere with participation - were psychotic or had a severe mental disorder

*ADL, Activities of Daily Living; BPSD, Behavioral and Psychological Symptoms of Dementia; DSM-5=Diagnostic and Statistical Manual of Mental Disorders-5; FAST, Functional Assessment Staging; NPI-NH, Neuropsychiatric Inventory-Nursing Home Version*.

### Measurements

Researchers involved in the DEM.LIGHT trial supervised nurses in the use of assessment tools. Only staff that knew the patients well, i.e., the regular nursing staff, working directly with the patients, completed the questionnaires. Daytime personnel completed the questionnaires used in the present study, as part of a larger data collection. The daytime nurses usually convey information about patients to the attending physician and are normally well-informed about nocturnal behavior of the patients. In the present study, the questionnaires were administered either the same week as the patients wore an actigraph or the following week. The questionnaires were completed once. Sociodemographic characteristics, medication status, and diagnoses were collected from medical records.

The Mini-Mental State Examination (MMSE) (31) was used to evaluate cognitive impairment at baseline. The total score ranges from 0 to 30; zero to ten points corresponds to severe dementia, 11–20 to moderate, 21–25 to mild dementia, 26–29 to questionable dementia, and 30 to no dementia (32). The MMSE was administered the same week as the patients wore the actigraph.

Sleep disturbance symptoms were assessed with the SDI, which evaluates nocturnal behavior for the last 2 weeks (30). The SDI is derived from the sleep item and its follow-up-questions of the Neuropsychiatric Inventory (NPI) (33). The NPI evaluates the frequency, severity, and caregiver distress of several behavioral and psychological disturbances which commonly occur in dementia, including disturbed sleep. The questions pertain to the previous 4 weeks. Each item (e.g., agitation/aggression, anxiety, sleep) has a description to aid determining whether and to what extent a disturbance occurs. For the sleep item the description includes: “Does the patient have difficulty sleeping? Is he/she up at night? Does he/she wander at night, get dressed, or disturb your sleep?” Endorsement of any of these behaviors elicits seven follow-up questions. The NPI sleep item score is based on a single frequency and severity rating for all the sleep disruption-related behaviors. The SDI was developed by assigning a frequency (0–4), severity (0–3), and caregiver distress (0–5) score to each of the follow-up questions of the NPI sleep item (30).

The SDI was developed to be rated by the live-in caregivers of home-dwelling seniors suffering from dementia. For the DEM.LIGHT trial, the SDI was translated to Norwegian and adapted to the nursing home context. Item 4 (“awakening you during the night”) was changed into (“awakening at night”), in order to take into account that patients may be awake at night without engaging in any of the behaviors covered by other items (e.g., wandering, getting out of bed). The translation process adhered to standard guidelines to reach a cultural equivalence of instruments (34). As some of the wording in the SDI was identical to the NPI, which had already been translated to Norwegian (35), we used the existing translations when possible. The SDI contains eight items (see Supplementary Material), where the eighth item asks about any additional information not captured by items 1–7. Only items 1–7 were included in the total score.

In the original paper, Tractenberg et al. (30) calculated the total SDI score as the average frequency multiplied by the average severity. This total score has been used by other authors adopting the scale (36, 37). When using this calculation, the total score may however vary greatly depending on the distribution of frequency and severity scores across items. For example, having three frequently occurring symptoms (frequency = 4) of mild severity (severity = 1) produces a higher total score (=0.74, calculations provided in the Supplementary Material) than having one frequently (frequency = 4) occurring symptom of marked severity (severity = 3; = 0.25). Other authors have therefore calculated the total score as the sum of the products of the frequency and severity of each of the single items of the scale (38, 39). This way of calculating the total score provides the same total score for both of the scenarios outlined above (both = 12). In the present study, both approaches to total score calculation were investigated, where the former is referred to as the “*SDI average total score*” and the latter is referred to as the “*SDI summed product score*.” We also calculated summed frequency as a general indicator of “overall disturbance” (referred to as the “*SDI summed frequency score*”). Higher values on all of these composite scores represent “worse sleep disturbance,” although as noted above, these summaries are not linearly comparable. In line with the original paper (30), mean frequency, severity, and caregiver distress were also calculated.

The single sleep item from the nursing home version of the NPI (NPI-NH) (35, 40) was investigated in relation to the SDI. The NPI-NH asks about the previous 4 weeks. The NPI-NH sleep item comes with a description similar to the NPI, followed by six follow-up questions (including “other nighttime behaviors”). Previous studies have suggested a cut-off product score of frequency (1–4) multiplied by severity (1–3) of ≥4 to define the presence of sleep disturbance on this single item from the NPI-NH (25, 41). In contrast to the SDI, the NPI-NH follow-up questions do not ask about excessive daytime sleep (SDI item 7) or if the patient wake up during the night without engaging in any specific behaviors (SDI item 4).

Sleep was objectively measured using the *Actiwatch II (Philips Respironics)*. Actigraphs are wrist-worn devices that can measure activity across several days, and even weeks (42). The actigraphs were placed on the dominant wrist, in accordance with previous studies in this population (8, 25). Each 1-min epoch was scored as either sleep or wake by the *Actiware 6.0.9 (Philips Respironics)* software, based on activity from the two epochs immediately preceding and following the relevant epoch. The threshold for wakefulness was set to medium. Activity data were collected for 7 days and patients had to complete at least five nights of recordings to be included in the analyses. We initially planned to score the actigraphic recordings manually, in line with a premediated scoring protocol (25). However, it was challenging to determine the start and end of the rest intervals. The event buttons were not consistently pressed by the nursing home staff. Additionally, many dementia patients have severely fragmented sleep, thus, there were rarely clear indications of bedtime and rise time in the actograms, normally indicated by a marked and sustained decrease/increase in activity and/or light levels (25, 43). These challenges are common in this population, and researchers have typically solved these issues by setting a fixed rest interval [e.g., (8, 14, 44–53)]. A range of rest intervals have been used earlier and a fixed rest interval from 22:00 to 06:00 was chosen for the present study, as it represents a sensible intermediate of these. It was expected that the majority of patients would be in bed by 22:00 and that the aforementioned interval would overlap with the main sleep episode of most of the participants. When using a fixed rest interval, some commonly reported actigraphy outcomes become invalid, such as sleep onset latency and early morning awakenings. Thus, the following actigraphy outputs were extracted from the rest interval: Sleep efficiency (SE, the percentage of time spent asleep in the rest interval), total sleep time (TST), and wake-after-sleep-onset (WASO, the time spent awake after sleep onset). While TST is a quantitative measure of sleep, SE and WASO reflect mainly sleep quality, although the latter parameters do not necessarily correspond with subjectively reported sleep quality (54). The scores used for SE, TST, and WASO were calculated as the mean value for all nights of recorded actigraphy. These outputs are largely linear in a fixed rest interval. In addition, the 24 h fragmentation index was extracted, as an indication of the overall disturbance of the sleep-wake rhythm across the day and night.

Having a SE of below 85% is often used as a cut-off for identifying disrupted sleep in otherwise healthy populations (55, 56). This corresponds to a TST of 6 h and 48 min in the fixed rest interval, which is close to the 7 h that is considered normal in healthy populations (57). Dementia patients frequently sleep during the day and some stay in bed for 12–13 h per day (25), and it was therefore considered too strict to use a cut-off of 6 h and 48 min TST in the present study. Thus, in agreement with Yesavage et al. (52), we used TST as the indicator of overall sleep disturbance and TST values <6.0 h were characterized as “disturbed” sleep, while those sleeping 6 h or more were characterized as having “not disturbed” sleep. This cutoff corresponds to a SE of 75% in the fixed rest interval (22:00–06:00), a cutoff that has previously been used in dementia populations (58).

### Statistical Analyses

Statistical analyses were performed in *SPSS for Windows, version 25.0*. All data were analyzed for normality and non-normal data were analyzed using non-parametric tests. Confidence intervals (CIs) for medians were calculated using the Ratio Statistic in SPSS. Due to the lack of distributional assumptions, the 95% CIs for the Ratio Statistic represent approximations.

#### Missing Data and Imputation

There were some missing data on the SDI at baseline. Little's MCAR test was not significant (*p* =0.151), meaning that data were missing completely at random (59). Imputations were thus made by Expectation Maximization (EM) when questionnaires were missing <20% of items (31 items from 11 patients, 2.2% of all items). Three questionnaires were missing ≥20% and data from these were excluded altogether from the analyses.

#### Internal Consistency

Internal consistency of the adapted SDI was evaluated using Cronbach's alpha (60), estimated as item-total correlations. A Cronbach's alpha of 0.7 and above is normally considered acceptable (61). The internal consistency analyses were computed separately for the frequency and severity ratings.

#### Convergent Validity

The strength of the relationships between the three different SDI total scores, the single NPI sleep item (frequency × severity), and actigraphic parameters were explored using Spearman correlations. As TST and SE are perfect linear functions of each other in a fixed rest interval, only TST, WASO, and fragmentation index were included in this analysis.

#### Test Accuracy

Receiver operating characteristic (ROC) curves were calculated for the actigraphy-based cut-off (“disturbed sleep” defined as a TST of <6 h) against the SDI outcomes, in order to investigate the diagnostic performance of each of the SDI composite scores (*SDI average total score, SDI summed product score*, and *SDI summed frequency*). We defined “disturbed sleep” as an average actigraphy TST value of <6 h; this dichotomous variable was the outcome in the ROC curve analysis (30, 52). The “area under the curve” (AUC) score reflects the discriminatory ability of the test (62) or SDI summary, in this case, for the outcome (disturbed sleep as defined by TST). A high AUC score implies that the rate of true positives is high and that the rate of false positives is low. An AUC score below 0.75 is not considered clinically useful (62).

Sensitivity, specificity, predictive values, and the rate of true positives, false positives, true negatives, and false negatives were calculated for the SDI summaries, to investigate which outcome and which cut-off was the most clinically useful. Sensitivity refers to the proportion with the condition that get a positive test result (true positives), while specificity refers to those who do not suffer from the condition and that get a negative test result (true negatives) (63). The positive predictive value refers to the proportion of positive results that are true positives, while the negative predictive value refers to the proportion of negative results that are true negatives.

#### Youden's Index

The Youden's index (sensitivity + specificity-1) is a common summary measure for the ROC curve and is used to determine the most appropriate cut-off value for a scale (64–66). This index incorporates sensitivity and specificity and the cut-off that yields the highest Youden's index value is regarded as the “optimal” threshold value. The Youden's Index was calculated for the *SDI summed product score* and the *SDI summed frequency score*.

### Ethical Approval and Consent to Participate

Through conversations with the physician at each nursing home, patients who were most likely able to provide informed consent were identified. The researchers endeavored to inform all participants about the study in an adapted way, and continuously evaluated the capacity to provide consent. Most patients were not able to provide consent. In these cases, the patient's legal guardian was contacted directly. After being approached by a phone call, they received a letter by postal mail containing all relevant information about the aims, proceedings, and ethical approval of the trial, after which they gave a presumed informed consent on behalf of the patient. In giving a presumed consent, the patient's guardian was instructed to consider what the patient would have wished for in this situation, not what they themselves believed was most pertinent. Across the study period, the researchers were sensitive to any expressions of discomfort or protests from the participants; and considered this as withdrawal of consent. The study was approved by the Regional Ethics Committee (REC South East 2016/2246).

## Results

**Figure 1:**
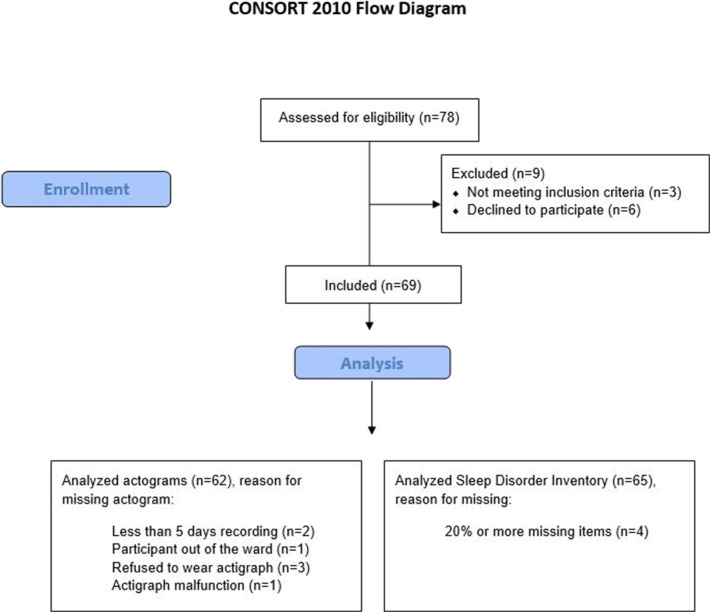
A flow chart of the inclusion process. Allocation to intervention group and placebo group omitted here as only baseline data are used in present study.

A total of 69 participants were enrolled, of whom 68% were women, mean age was 83.5 (SD 7.1), and mean MMSE was 6.4 (SD 6.7). Descriptive statistics and diagnoses are provided in Table 2. Figure 1 shows the full inclusion and exclusion of study participants leaving 62 with actigraphy recordings over at least 5 days and 65 with completed SDIs. A total of 59 patients had both completed SDI and had sufficient actigraphy recordings.

**Table 2:** Descriptive statistics for the 69 patients.

Age (mean, SD)	83.5 (7.1)
Female (%)	68.0%
Mini mental state examination sum score, mean (SD) (*n* = 56)	6.4 (6.7), median 4.0
Dementia diagnoses, *n* (%)	
Alzheimer's disease (AD)	38 (55.1)
Mixed AD and vascular dementia	0
Lewy body dementia	1 (1.4)
Other dementia	2 (2.9)
Vascular dementia	4 (5.8)
Frontotemporal dementia	0
Parkinson's dementia	0
Unknown dementia	21 (30.4)
No diagnosis^¤^	3 (4.4)
Neuropsychiatric inventory (NPI) (*n* = 69)	
NPI total score, median (25th−75th percentile)	21.0 (6.0–42.0)
Sleep item score, median (25th−75th percentile)	0.0 (0.0–4.0)
Sleep item score ≥ 4, *n* (%)	18 (26.1)
Sleep item score 0, *n* (%)	38 (55.1)
Total number of medications (mean, SD)	6.7 (2.8)
Number of psychotropic medications^*^ (mean, SD)	2.9 (1.3)
Number of sedatives and hypnotics^§^, *n* (%)	
0	60 (87.0)
1	8 (11.6)
2	1 (1.4)

* All medications with an ATC code starting with N.

§ All medications with an ATC code starting with N05C.

¤ *These patients were still included as their scores on the Mini Mental State Examination and the Functional Assessment Staging suggest moderate and severe dementia. In addition, clinically trained researchers concluded that they with high probability suffered from dementia according to the DSM-5 criteria*.

### Sleep Assessed With SDI

The SDI scores were not normally distributed, with the majority of patients obtaining low scores. Therefore, the median was used to summarize the group, instead of the mean (Table 3). In all, 19 patients (32%) had a total score of 0. The median of the *SDI average total score* was 0.06 and the median of the *SDI summed product score* and *the SDI summed frequency score* was 3.00. Mean frequency was 0.43, mean intensity was 0.14, and mean staff distress was 0.29. All SDI-based scores had a wide range, reflecting heterogeneity in the sample.

**Table 3:** Median values for the SDI outcomes for the 59 patients that had both SDI and actigraphy data, all of which had non-normal distributions.

**SDI outcome (possible min-max score)**	**Median**	**25th percentile**	**75th percentile**	**Min-max**	95% CI of median^*^
SDI average total (0–12)	0.06	0.00	1.84	0.00–6.12	0.04–0.57
SDI summed product (0–84)	3.00	0.00	18.00	0–60	2.00–9.00
SDI summed frequency (0–28)	3.00	0.00	11.00	0–22	2.00–7.00
SDI mean frequency (0–4)	0.43	0.00	1.57	0–3	0.29–1.00
SDI mean severity (0–3)	0.14	0.00	0.86	0–2	0.14–0.57
SDI mean distress (0–5)	0.29	0.00	1.43	0–4	0.00–0.86

SDI, Sleep Disorder Inventory.

* *CI for medians were calculated with the Ratio Statistic in SPSS, which provide varying coverage, but always more than 95%*.

The most frequent behavior was waking up at night, happening at least once a week (i.e., endorsed by the staff) for 46% of the patients (Table 4). Getting up during the night was endorsed for 34% of the patients and 31% engaged in wandering or inappropriate activities during the night. The three items reflecting these behaviors were also the most distressing to the nursing personnel. One third of the patients were reported to sleep excessively during the day at least once a week, but this behavior caused less distress among staff.

**Table 4:** The endorsement of each SDI item (frequency ≥2), percentage rated as being moderately to markedly severe (severity ≥2), and percentage rated as moderately to extremely distressing (staff distress ≥3), in accordance with Tractenberg et al. (30).

**Symptom**	**% Endorsement**	**% Moderate/** **marked severity**	**% Moderate/** **extreme distress**
Difficulty falling asleep	25.43	16.95	13.56
Getting up during the night	33.90	27.12	25.42
Wandering, pacing or getting involved in inappropriate activities at night	30.51	23.73	25.42
Awakening at night	45.76	30.51	23.73
Awakening at night, dressing, and planning to go out, thinking that it is morning and time to start the day	11.86	8.48	6.78
Awakening too early in the morning (earlier than is his/her habit)	8.48	3.39	1.70
Sleeping excessively during the day	33.90	13.56	3.39

SDI, Sleep Disorder Inventory.

*Frequency ratings: 0 = not present; 1 = less than once per week; 2 = 1–2 times per week; 3 = several times per week but less than every day; 4 = once or more per day (every night). Severity ratings: 0 = not present; 1 = mild; 2 = moderate; 3 = marked. Staff distress ratings: 0 = not at all; 1 = minimally; 2 = mildly; 3 = moderately; 4 = severely; 5 = very severely/extremely (30)*.

### Sleep Assessed With Actigraphy

The actigraph results were based on recordings including a mean of 7.6 (SD 1.4, range 5–14) nights. The median sleep length (TST) within the rest interval (i.e., at night) was 6 h 19 min (95% CI 5 h 23 min−6 h 41 min) and the median SE was 79% (95% CI 69–84) (Table 5). The time spent awake after sleep onset (WASO) was normally distributed and had a mean of 1 h 9 min (95% CI 57 min−1 h 20 min). The 24 h fragmentation index had a mean of 93.2 (95% CI 88.0–98.3). All actigraphy outcomes had a wide range, reflecting heterogeneity in the population.

**Table 5:** Actigraphy variables for the 59 participants with complete SDI and actigraphic recordings based on a mean of 7.6 days.

	**Mean (SD)**	**Range**	**95 % CI^**§**^**
TST night	379.10 (290.86–423.00)^*^	123.00–463.40	323.57–401.00
SE	79.00(60.60–88.13)^*^	25.63–96.54	68.59–83.54
WASO	68.47 (43.72)	3.00–212.71	57.08–79.87
24 h fragmentation index	93.15 (19.58)	61.03–141.63	88.04–98.25

SE, sleep efficiency; TST, total sleep time; WASO, wake after sleep onset.

* *Non-normal data presented as medians with the 25th and 75th percentile in parentheses. ^§^CI for medians were calculated with the Ratio Statistic in SPSS, which provide varying coverage, but always more than 95%*.

### Internal Consistency of the SDI

Cronbach's alpha for the adapted SDI was 0.82 for the frequency ratings and 0.87 for the severity ratings. The item-total correlations varied across items and were below 0.3 for the frequency of item 7 (excessive sleep during the day;0.22), the severity of item 6 (wake up too early;0.23) and the severity of item 7 (excessive sleep during the day;0.25). Removing these items caused only negligible increases of alpha. Daytime sleep propensity became more severe with increasing AD severity (67), hence the daytime sleep item (item 7) provided relevant clinical information; thus even if item-total correlations are lowest for these items/ratings, they are a clinically-essential component of the instrument and its scores.

### Convergent Validity: SDI Compared to Actigraphy and NPI-NH

Table 6 shows the Spearman correlation coefficients between different SDI variables and the actigraphy parameters. For TST at night, all SDI outcomes had a significant and moderate correlation (minimum −0.40 and maximum −0.44). As expected, greater SDI scores were associated with lower TST (resulting in a negative correlation), higher WASO, and higher scores on the single NPI-NH sleep item (positive correlations). The SDI summaries did not correlate significantly with the 24 h fragmentation index.

**Table 6:** The correlation coefficients (Spearman's rho) for the SDI outcomes against actigraphy outcomes and the NPI-NH sleep item.

**SDI outcome**	**TST night** **(actigraphy)**	**WASO** **(actigraphy)**	**24 h fragmentation** **index (actigraphy)**	**NPI-NH sleep item**
SDI average total	−0.431^*^	0.389^*^	0.216	0.746^*^
SDI summed product	−0.432^*^	0.402^*^	0.216	0.751^*^
SDI summed frequency	−0.436^*^	0.395^*^	0.213	0.754^*^
Mean frequency	−0.436^*^	0.395^*^	0.213	0.754^*^
Mean severity	−0.403^*^	0.369^*^	0.195	0.749^*^
Mean distress	−0.408^*^	0.372^*^	0.160	0.755^*^

NPI-NH, Neuropsychiatric Inventory—nursing home version; SDI, Sleep Disorder Inventory; TST, total sleep time; WASO, wake after sleep onset.

* *Correlations were significant at the 0.01 level (2-tailed)*.

### Test Accuracy

As noted, we defined “disturbed sleep” to be actigraphy-derived TST of <6 h for the ROC curve analysis (1 = TST at night <6 h, 0 = TST at night ≥6 h). Twenty-seven patients had a TST below 6 h, and 32 patients had a TST of 6 h or more. We evaluated how the three SDI summaries performed against this standard (Table 7). The AUC scores were above 0.75 for all three SDI summaries, indicating that all are clinically useful. The scores were almost equivalent, however, the *SDI summed product score* and the *SDI summed frequency score* both had slightly higher AUC scores than the *SDI average total score*, with AUC scores of 0.78, 0.78, and 0.77, respectively.

**Table 7:** The ROC output for the SDI summaries against the 6 h actigraphy TST cut-off.

				**95% CI**	
**SDI outcome**	**Area**	**Std. error**	**Asymptotic sig**	**Lower** **bound**	**Upper** **bound**
Total *SDI average total*	0.771	0.064	0.000	0.646	0.895
Total *SDI summed product*	0.777	0.063	0.000	0.653	0.900
Total *SDI summed frequency*	0.780	0.062	0.000	0.659	0.901

*CI, confidence interval; ROC, Receiver operating characteristic; SDI, Sleep Disorder Inventory; TST, Total sleep time*.

The sensitivity, specificity, predictive values, and ratios of true positives, false positives, true negatives, and false negatives were calculated for each level of the *SDI summed product score* (range 1–84) (Table 8) and the *SDI summed frequency score* (range 1–28) (Table 9). For both the *SDI summed product score* and the *SDI summed frequency score*, Youden's index peaked at cut-off scores of 5–6 and the results are presented for values 1–10. The sensitivity, specificity, and Youden's Index of the *SDI average total score* (not shown) were all worse than the two best AUC performing summaries.

**Table 8:** The sensitivity, specificity, positive and negative predictive values, and the rate of true positives, false positives, true negatives, false negatives, and the Youden's Index for each value of the *SDI summed product score* (sum of frequency × severity).

**SDI summed product score**	**Sensitivity (%)**	**Specificity (%)**	**PPV (%)**	**NPV(%)**	**TP**	**FP**	**TN**	**FN**	**Youden's Index**
≥1	85	47	58	79	23	17	15	4	0.321
≥2	85	47	61	81	23	15	17	4	0.321
≥3	78	63	64	77	21	12	20	6	0.403
≥4	74	72	69	77	20	9	23	7	0.460
≥5	70	78	73	76	19	7	25	8	0.485
≥6	70	78	73	76	19	7	25	8	0.485
≥7	63	81	74	72	17	6	26	10	0.443
≥8	63	81	74	72	17	6	26	10	0.443
≥9	63	81	74	72	17	6	26	10	0.443
≥10	59	84	76	71	16	5	27	11	0.437

*Total n = 59. FN, false negative; FP, false positive; NPV, negative predictive value; PPV, positive predictive value; SDI, Sleep Disorder Inventory; TN, true negative; TP, true positive*.

**Table 9:** The sensitivity, specificity, positive, and negative predictive values, and the rate of true positives, false positives, true negatives, false negatives, and the Youden's Index for each value of the *SDI summed frequency score*.

**SDI summed frequency score**	**Sensitivity (%)**	**Specificity (%)**	**PPV (%)**	**NPV (%)**	**TP**	**FP**	**TN**	**FN**	**Youden's Index**
≥1	85	47	58	78	23	17	15	4	0.321
≥2	85	53	61	81	23	15	17	4	0.383
≥3	78	63	64	77	21	12	20	6	0.403
≥4	70	75	70	75	19	8	24	8	0.454
≥5	67	81	75	74	18	6	26	9	0.480
≥6	67	81	75	74	18	6	26	9	0.480
≥7	63	84	77	73	17	5	27	10	0.474
≥8	59	84	76	71	16	5	27	11	0.477
≥9	56	84	75	69	15	5	27	12	0.400
≥10	48	88	77	67	13	4	28	14	0.357

*Total n = 59. FN, false negative; FP, false positive; NPV, negative predictive value; PPV, positive predictive value; SDI, Sleep Disorder Inventory; TN, true negative; TP, true positive*.

Comparing the *SDI summed product score* and the *SDI summed frequency score*, the former achieved the highest Youden's index (0.485 compared to 0.480). These values were obtained for both a cut-off of ≥5 and a cut-off ≥6, for both the *SDI summed product score* and *the SDI summed frequency score*. For the *SDI summed product score*, these cut-offs had a sensitivity of 70%, a specificity of 78%, a positive predictive value (PPV) of 73% and a negative predictive value (NPV) of 76%. For the *SDI summed frequency score*, these cut-offs yielded a sensitivity of 67%, a specificity of 81%, a PPV of 75%, and a NPV of 74%.

## Discussion

The aim of the present study was to validate the SDI in a nursing home context and to determine a clinically useful cut-off score on the SDI to identify sleep disturbance in this population. The analyses showed that the SDI had high internal consistency and convergent validity.

Even though two items had low item-total correlation, they were not excluded because they minimally affected the overall internal consistency and thus may provide important clinical information. Three different ways of summarizing the SDI correlated significantly with the actigraphy outcomes TST at night and WASO, with medium-strength associations. Considering frequency, severity, and staff distress separately, frequency had the strongest association to these actigraphy sleep variables. The SDI summaries did not correlate with 24 h sleep fragmentation. Although the SDI contains one item pertaining to daytime sleep, the total score did not seem to reflect the fragmentation of the sleep-wake rhythm across the day and night. The ROC curve analyses indicated that the *SDI average total score*, the *SDI summed product score*, and the *SDI total frequency score* led to correct predictions of disrupted sleep (yes and no) about 78% of the time (95% CI about 65–90%), which is considered to be “clinically useful” (62). The *SDI summed product score*, using a cut-off for disturbed sleep of five or more or six or more had the highest Youden's Index values. Both cut-offs yielded a sensitivity of 70%, a specificity of 78%, a positive predictive value (PPV) of 73% and a negative predictive value (NPV) of 76% for predicting disturbance defined as <6 h in TST defined by actigraphy. The *SDI summed frequency score* had the highest AUC score, however, obtained a slightly lower maximum Youden's index. The maximum Youden's index on this summary was also obtained by both a cut-off of five or more and six or more, yielding a sensitivity of 67%, a specificity of 81%, a PPV of 75%, and a NPV of 74%. Because it is important to be as sensitive to disrupted sleep as possible, we believe that the SDI summary providing the highest sensitivity should be used (i.e., the *SDI summed product score*), and also that the lower cut-off (≥5) should be used. Thus, we suggest a clinical cut-off for the presence of disturbed sleep in institutionalized dementia patients to be a *SDI summed product score* of five or more. Even though the SDI was developed for home-dwelling seniors and their caregivers (30), the present study demonstrates that the SDI can be clinically useful for the identification of sleep disturbance when administered by daytime staff in a nursing home context.

The present finding of the clinical utility of a proxy-rated sleep tool stands in contrast to the findings by Blytt et al. (25), where the sleep items of the CSDD and the sleep item from the NPI-NH underreported sleep problems when compared to actigraphy parameters. Other than using different subjective sleep outcomes, the discrepancy between the present results and the results from Blytt et al. (25) may in part be explained by differences in the choice of actigraphy-based cut-offs for defining disturbed sleep. In the present study, disturbed sleep was defined as sleeping <6 h between 22:00 and 06:00, corresponding to a SE of 75% or less. More conservatively, Blytt et al. (25) defined disturbed sleep as having <85% SE, in each participants' individual rest interval (based on light, activity, and event marker information in the actogram, indicating bedtime and wake time), which is a common cut-off for defining disturbed sleep (55, 56), albeit in normal populations.

In the original paper by Tractenberg et al. (30), they defined a rest interval from 20:00 to 08:00. To avoid the inclusion of time spent out of bed, we instead used a rest interval from 22:00 to 06:00, reflecting a more realistic interval in this specific population. The use of a common night-time interval for all participants in our sample reflects a period when patients are expected to be in bed. Using a wider rest interval might have increased the variability of SDI ratings across wards or nurse raters, as problematic night-time behavior in one ward could, for example, be classified as afternoon or evening restlessness in a ward with later bed time. Conversely, a narrow rest interval may have excluded time when many patients were in bed and asleep, as nursing home patients spend substantial time in bed (25).

In the original study, Tractenberg et al. (30) found a higher prevalence of symptoms than in the present study. However, in their study, participants were recruited based on sleep complaints, while disrupted sleep was only one of several optional inclusion criteria in the present study. Also, bed-sharing caregivers [as in (30)] are likely to be more sensitive to nocturnal behavior than nursing home staff, as staff generally do not attend to the patients at night unless they get up or call for assistance. Further, sleep difficulties or nocturnal behavior might not be reported consistently in the patient records and may not always be conveyed to the day shift staff. This might explain the low clinical cut-off suggested in the present study: A sum frequency of five is low, given that the maximum score is 84. This suggests that even this slight subjective impression of sleep disturbance among patients on the nursing home staff, may reflect a significant disruption of sleep. However, we did observe that the distributions of SDI summary scores were skewed toward the low-scoring end of the continuum, and 44% of the sample would be qualified as “sleep disturbed” using the cut-off of 5, as compared with 46% characterized as “sleep disturbed” using the clinical and objective TST cut-off of <6 h.

The present study suggests that the SDI, rated by daytime staff, may be used to detect sleep problems in institutionalized dementia patients. The cut-off score identified can be used as a means of identifying patients for inclusion in clinical trials of sleep interventions. To treat disturbed sleep, it is necessary to identify the underlying cause. In line with this, the SDI can serve as a screening tool to identify patients who struggle with sleep problems, and form the basis for a more deliberate mapping of sleep problems. For example, sleep disturbance caused by nocturia requires a different treatment than sleep disordered breathing. Hoeckert et al. (27) used the CSINAPS scale, that specifically asked about snoring, breathing problems and unusual movements. These items were rarely endorsed despite high prevalence of sleep disordered breathing and periodic leg movements in dementia patients (68), reflecting the need to more deliberately evaluate these symptoms. Importantly, the use of objective measurements presents a challenge as many patients struggle with tolerating the equipment (69). Thus, deliberate and continuous observation of patients that struggle with sleep, for example patients identified by the SDI, is probably necessary in order to identify the specific underlying problems. However, the routine use of validated sleep scales may encourage the awareness among staff of how clinically relevant sleep problems in these patients are, increasing staff sensitivity to the importance of detecting signs of poor sleep as a significant component in understanding the patient's overall behavioral problems.

### Strengths and Limitations

The majority of the included participants provided good quality actigraphy data for a minimum of 5 nights (mean 7.6 nights). The patients who agreed to wear the actigraph generally wore it continuously until it was collected by the researchers. Because the presence of disturbed sleep was not a required criterion for inclusion, the participants exhibited a wide range in scores on the sleep parameters. The present study demonstrated the utility of the SDI in a heterogeneous sample that is representative of institutionalized patients suffering from dementia.

One important limitation is the choice of an actigraphy-based outcome as the reference against which the SDI was validated. Wrist actigraphy has been shown to be a reliable method of assessing sleep in different clinical populations, compared to the “gold standard” of polysomnography and observation (70), including nursing home patients with severe dementia (69). However, studies have demonstrated that actigraphy has low specificity (poor wake detection) (71) and that it overestimates sleep in people with very disturbed sleep (8, 72). Thus, actigraphy has acknowledged weaknesses in terms of detecting wakefulness, hence the correlations between SDI summaries and actigraphic data found in the present investigation might represent overestimates. Another limitation is the suboptimal use of the event marker and consequently the use of a fixed rest interval. Future studies should secure a robust indication of bedtime and rise time to obtain more accurate reports of each participants' sleep.

Further, even though the ROC curve analysis revealed a clinically useful cut-off for the SDI summaries, the AUC scores of 0.77 (*SDI average total*) and 0.78 (*SDI summed product, SDI summed frequency*) still correspond to a relatively low discriminatory power (62). The confidence intervals for the AUC scores were quite wide and ranged from about 0.65, corresponding to no clinical value, to 0.90, corresponding to high clinical value (62). This indicates somewhat uncertainty about value of the SDI summaries. We thus suggest that the findings should be replicated in a larger sample.

The correlation between the SDI summed product score and actigraphically-measured TST was 0.43, corresponding to a moderate correlation (73), but also shows that there is significantly residual variance in TST not captured by the SDI. In fact, the amount of shared variability was <20% (0.43 ×0.43 = 0.185) and over 80% of the variability in SDI scores was not explained by the TST value. As the actigraphy-based TST was a summary across a minimum of 5 days (mean 7.7), and the SDI is a summary across 2 weeks, there was not an exact temporal overlap between the two measures. The SDI covered the last 14 days and was completed in the same week that the actigraph was worn by the participants. Hence, the temporal overlap between the two measures was incomplete, and thus the SDI includes behavior not captured by the actigraph. Importantly, there is however a well-documented discrepancy between subjective and objective measures of sleep (74, 75), and this discrepancy is probably a strong contributor to the residual variance in actigraphically measured TST in the present study, in addition to the lack of temporal correspondence. Further, there is a lot more complexity to “disrupted sleep” than what is captured by actigraphically assessed TST alone, which is, at its core, a reflection of immobility. However, both outcomes (actigraphy-measured TST and the SDI summary) were used as approximations of general sleep disturbance, where both can serve as indicators that a more deliberate evaluation of sleep is warranted.

The completion of the SDI was part of a larger data collection project and all questionnaires were completed by nurses during the day. Few nurses are at work during the night and our data collection corresponds with clinical assessments in nursing homes, which are normally performed during the day in collaboration with the nursing home physician. The night time staff are obliged to write reports and to note in the medical record if clinically relevant events have taken place during night shifts. It is also common that staff share information orally during handover. Thus, daytime staff should be informed about any nocturnal events. It would however be preferable to obtain both night and daytime staff reports on the SDI. Nevertheless, the fact that we did achieve an AUC score of 0.78 and significant correlations between the SDI and actigraphy demonstrates that daytime staff have significant information about patients' behavior outside their own shift. It also suggests that the usual procedure of administering tests during the day is feasible also with the SDI, provided adequate communication between the night and day shift.

While the *SDI summed product score* and the *SDI summed frequency score* both led to the same cut-off value (≥5) for identifying disrupted sleep, the sample size of the present study was relatively small and confirmatory studies in larger samples are warranted. We did not interrogate the specific diagnoses or prescriptions of sedatives of the participants included in the trial, and there may be important differences among those with AD, dementia with Lewy bodies, and vascular dementia. Future studies should address these issues.

## Conclusion

Overall, the results of the present study showed that the scores on the proxy-rated Sleep Disorder Inventory correspond well to objectively measured sleep disruption (defined as a TST <6 h, corresponding to a SE <75%) in institutionalized dementia patients, using a clinical cut-off of a summed product score of five or more. The present results should be interpreted with caution bearing in mind that actigraphy was used as the reference outcome measure of sleep. Even though the SDI seems to identify patients with disturbed sleep successfully, some patients suffering from disturbed sleep may still go undetected. The recommended cut-off score (≥5) is low, suggesting that only a slight clinical impression of disrupted sleep may reflect significant sleep disruption. Nursing home staff should be vigilant to document any signs of sleep problems among patients at all times. The SDI appears to be useful as a screening tool to identify patients with probable sleep problems. However, determining the cause of the disrupted sleep normally would require a more deliberate approach, such as continuous observation and/or polysomnografic/polygraphic recordings.

## Data Availability Statement

The datasets generated for this study are available on reasonable request to the corresponding author.

## Ethics Statement

The studies involving human participants were reviewed and approved by the Regional Ethics Committee (REC South East 2016/2246). The patients/participants provided their written informed consent to participate in this study.

## Author Contributions

GH, EK, SP, EF, ET, IN, and RT were involved in the acquisition or analysis of the data. All authors were involved in the interpretation, drafting, revision of the work, approved the manuscript for submission, agreed to be accountable for the work, and contributed in the conception or design of the work.

### Conflict of Interest

The authors declare that the research was conducted in the absence of any commercial or financial relationships that could be construed as a potential conflict of interest.
