# Supplementary material for: An Actigraphy-Based Validation Study of the Sleep Disorder Inventory in the Nursing Home
Source: Front Psychiatry. 2020 Mar 13;11:173. doi: 10.3389/fpsyt.2020.00173 (PMC7083107; doi:10.3389/fpsyt.2020.00173)
Supplement: Supplementary file 1 [file Data_Sheet_1.docx]

Supplementary Material

# The sleep Disorder Inventory (SDI) for the nursing home setting

| Symptom |
| --- |
| 1. Difficulty falling asleep |
| 1. Getting up during the night (do not count if the subject gets up once or twice per night to go to the bathroom and quickly falls back to sleep) |
| 1. Wandering, pacing or getting involved in inappropriate activities at night |
| 1. Waking up during the night |
| 1. Awakening at night, dressing, and planning to go out, thinking that it is morning and time to start the day |
| 1. Awakening too early in the morning (earlier than is his/her habit) |
| 1. Sleeping excessively during the day |
| 1. Other night-time behaviors that are troublesome |

Frequency:

0: Not present in the last 2 weeks

1: Less than once per week

2: One to two times per week

3: Several times per week but less than every day

4: Once or more per day (every night)

Severity:

0: Not present

1: Mild: night-time behaviors occur but are not particularly disruptive

2: Moderate: night-time behaviors occur and disturb the patient and the sleep of the caregiver; more than one type of night-time behavior may be present

3: Marked: night-time behaviors occur; several types of night-time behavior may be present; the patient is very disturbed during the night

Occupational disruptiveness: How much does this behavior upset you and/or create more work for you?

0: Not at all

1: Minimally

2: Mildly

3: Moderately

4: Severely

5: Very severely or extremely

# Scoring rules

***SDI average total score:*** The average frequency of item 1-7 multiplied with the average severity of item 1-7. Minimum score 0, maximum score 12.

Example of total score calculation:

Three frequently occurring symptoms of mild severity (and four symptoms with a frequency of zero): ((4+4+4+0+0+0+0)/7) x ((1+1+1+0+0+0+0)/7)=0.74

One frequently occurring symptoms of severe severity (and six symptoms with a frequency of zero): ((4+0+0+0+0+0+0)/7) x ((3+0+0+0+0+0+0)/7)=0.25

***SDI summed product score*:** The sum of the products of the frequency and severity of item 1-7. Minimum score 0, maximum score 84.

Example of total score calculation:

Three frequently occurring symptoms of mild severity (and four symptoms with a frequency of zero): ((4x1)+(4x1)+(4x1)+(0x0)+(0x0)+(0x0)+(0x0))=12

One frequently occurring symptoms of severe severity (and six symptoms with a frequency of zero): ((4x3)+(0x0)+(0x0)+(0x0)+(0x0)+(0x0)+(0x0))=12.
